# Correlation between vitamin D level and left ventricular myocardial function demonstrated by speckle tracking echocardiography among β-thalassemia major children

**DOI:** 10.1007/s00431-025-06250-1

**Published:** 2025-06-16

**Authors:** Eman Gamal Abdelrahman, Ahmed Mahmoud Ezzat, Lina Abdelhady Mohammed, Reem Zaki Mohamed Fakher, Samar Mahmoud Elbahy

**Affiliations:** 1 Pediatric Department, Faculty of Medicine, Benha University, Benha, Egypt; 2 Biochemistry Department, Faculty of Medicine, Benha University, Benha, Egypt

**Keywords:** β-Thalassemia major, Vitamin D, Speckle tracking echocardiography, Global longitudinal strain

## Abstract

Vitamin D deficiency is a recognized complication of β-thalassemia major (β-TM), impacting cardiac function. Speckle tracking echocardiography (STE) offers a sensitive assessment of myocardial deformation, particularly global longitudinal strain (GLS). This study aimed to assess the association between vitamin D levels and LV function in β-TM children and to evaluate the impact of vitamin D supplementation on cardiac parameters. Seventy-five β-TM children underwent vitamin D level assessment, conventional echocardiography, and STE. Patients with vitamin D deficiency/insufficiency (25-OHD3 < 30 ng/ml) received vitamin D supplementation (4000–5000 IU/day). Follow-up assessments were conducted after vitamin D normalization. Vitamin D insufficiency (81.3%) and deficiency (18.7%) were prevalent. Vitamin D levels were inversely correlated with age and disease duration (*p* < 0.001) and positively correlated with transfusion/chelation therapy onset. Conventional echocardiography showed an inverse correlation between vitamin D level and left ventricular end diastolic diameter (*p* < 0.001) and deceleration time (*p* = 0.003). STE revealed a positive correlation between vitamin D and GLS (*p* < 0.001). Vitamin D supplementation significantly increased median vitamin D levels (from 16.0 to 39.0 ng/ml, *p* < 0.001) and improved STE parameters, including AP4L, AP3L, AP2L, and GLS (*p* < 0.001), indicating enhanced myocardial function. *Conclusion*: Vitamin D deficiency in β-TM children was correlated with impaired cardiac function. Vitamin D supplementation significantly improved cardiac function. Regular monitoring and maintenance of adequate vitamin D levels are crucial for preventing adverse cardiac effects.

**Table:** 

**What is Known:** • *β-Thalassemia major is frequently complicated by cardiac dysfunction, a major contributor to mortality*. • *Cardiac iron overload is a primary driver of cardiac dysfunction in β-TM*.
**What is New:** • *Vitamin D deficiency leads to impaired cardiac function, beyond iron overload, in β-TM children*. • *Vitamin D supplementation could improve cardiac function in β-thalassemia major patients*.

**What is Known:**

• *β-Thalassemia major is frequently complicated by cardiac dysfunction, a major contributor to mortality*.

• *Cardiac iron overload is a primary driver of cardiac dysfunction in β-TM*.

**What is New:**

• *Vitamin D deficiency leads to impaired cardiac function, beyond iron overload, in β-TM children*.

• *Vitamin D supplementation could improve cardiac function in β-thalassemia major patients*.

## Introduction

Thalassemia, a hereditary hematological disorder, features impaired globin chain biosynthesis, causing persistent anemia and related complications [1]. Global prevalence varies, with higher rates in the Middle East and Asia [2]. In 2021, there were 1,310,407 cases of thalassemia worldwide [3]. The complex pathophysiology of β-thalassemia involves deletions or mutations of the β-globin gene [4]. This disrupts the synthesis of α/β-globin chain, leading to hemolysis, inadequate erythropoiesis, and complications like iron overload and bone abnormalities [5].

Frequent transfusions cause iron overload over time, leading to excessive iron buildup in vital organs, involving the heart, liver, and endocrine glands [6]. Iron excess produces structural alterations in the myocardium that could result in heart failure, a leading cause of death for thalassemia cases**.** Therefore, iron chelation therapy has significantly enhanced patients’ prognosis [7].

Iron buildup in the liver and skin disturbs the hydroxylation and synthesis process of 25-hydroxyvitamin D (25-OH vitamin D), resulting in vitamin D deficiency [8]. Diminished vitamin D impairs cardiac contractility and increases parathyroid hormone (PTH) [9]. Through the downstream activities of G protein-coupled receptors, the PTH directly impacts the myocardium and is linked to left ventricular hypertrophy, muscular weakening, and a decline in cardiac function [10]. Furthermore, vitamin D can impact heart function via controlling the renin-angiotensin-aldosterone system. In vitro, activated vitamin D inhibits the expression of the renin gene and controls the growth and division of cardiomyocytes [11].

Conventional echocardiography is not quite sufficient to early detect cardiac iron overload. Speckle tracking echocardiography (STE), a sensitive, non-invasive ultrasound imaging technique, tracks the “speckles” displacement in 2D-echocardiographic images in an angle-independent manner to evaluate the myocardial longitudinal deformation and provide a helpful regional functional assessment of myocardial segments during the cardiac cycle [12, 13]. STE can evaluate mild left ventricular (LV) myocardial dysfunction using strain and strain rate measures [14].

The use of STE has become increasingly vital and has been incorporated into diagnostic guidelines for various pathologies [15]. Its high accuracy, reproducibility, and feasibility have been broadly verified [16]. This study was conducted to assess the impact of vitamin D levels on LV myocardial function in children with β-thalassemia major (β-TM) using both conventional echocardiography and STE.

## Materials and methods

### Study population

This prospective study was carried out on 75 children with β-TM from the hematology outpatient clinic of the pediatric department at Benha University Hospitals from April 2023 to March 2024. Children with β-TM between 5 and 18 years who were on regular blood transfusions, maintaining their pretransfusion hemoglobin above 8 g/dl, and were taking regular iron chelation medications for >1 year were enrolled in this study. Children < 5 years, those who had taken vitamin D supplements within 6 months before the study, and those who had not been receiving regular transfusion or iron chelation for the last year were all excluded, in addition to children with congenital heart, liver, and renal diseases or type-1 diabetes mellitus. Every patient underwent clinical examination and laboratory tests, including vitamin D level testing, and imaging studies. Vitamin D was considered deficient if the level was < 20 ng/ml, insufficient if the level was 20–29.9 ng/ml, and sufficient if the level was ≥ 30 ng/ml [17]. Patients with insufficient/deficient vitamin D levels (< 30 ng/ml) were given vitamin D at a dose of 4000–5000 IU/day for 3 months [18]. Then, vitamin D level was measured again after three months. If the vitamin D concentration remained low, treatment was continued for an additional 3 months, and the vitamin D level was reassessed. When the vitamin D level was normal, patients were re-evaluated by imaging studies.

### Clinical examinations and lab investigations

A full history was obtained from each patient, involving the age at diagnosis, the age at the initial blood transfusion and the frequency of transfusions, the type and duration of chelation therapy and patient compliance to it, the presence of any cardiac symptom or any thalassemic complication, a history of drug intake, and previous operations such as splenectomy. All the children underwent a general examination, as well as detailed abdominal and cardiac examinations.

All participants had blood drawn for complete blood count, as well as for liver enzymes and renal function tests. Serum ferritin levels were measured by an enzyme-linked immunosorbent assay (ELISA), and patients were sorted into three groups based on their serum ferritin concentrations (< 2500 µg/L, 2500–5000 µg/L, and > 5000 µg/L). The chemiluminescent immunoassay (CLIA) technique was employed to analyze serum vitamin D levels [19].

The severity of thalassemia was assessed based on a severity scoring system composed of baseline hemoglobin level, the age at the first blood transfusion, the requirements for transfusion, and the size of the spleen; patients were given a score from 0 to 2 in each point, and then the severity of thalassemia was classified according to the total score into three categories, mild (scored 0–3.5), moderate (scored 4–7), and severe (scored 7.5–10) [20].

### Imaging studies

Conventional echocardiography, tissue Doppler imaging, and STE were used to assess each patient’s LV function and were performed 24 h following blood transfusion to minimize the impact of severe anemia on cardiac function. Patients were scanned using a General Electric^®^ Vivid 95 cardiac ultrasound machine. To provide the best possible visualization of the myocardium, a locally optimized preset protocol was used, and additional image optimization was performed for every patient in accordance with the British Echocardiography Society Guidelines [21].

STE evaluates myocardial strain, or regional deformation, in the longitudinal, radial, and circumferential directions. Compared to the parasternal pictures used for radial or circumferential assessment, global longitudinal strain (GLS) uses apical images, which are easier and more reproducible to obtain. The apical position and the conventional long-axis, two-chamber, and four-chamber images are obtained. Every view takes into account six myocardial segments, and each strain curve reflects the average strain value of one myocardial segment. The average of all peak systolic strain readings is used to compute GLS [22]**.**

### Ethical considerations

The study followed the Declaration of Helsinki and later amendments to research involving human participants. This study has been accepted by the Benha University Ethics Committee in Egypt (MoHP No. 0018122017/Certificate No. 1017), study number MS 23-3-2023. The patients’ legal guardians provided their informed permission.

### Statistical analysis

Statistical Package for the Social Sciences. (SPSS) Software version 25.0 was employed for statistical analysis. Data normality was examined via Kolmogorov-Smirnov/Shapiro-Wilk tests. Numerical data were presented as mean and standard deviation (SD) or median (range) and categorical data as frequency/percentage. Chi-square/Fisher-Exact tests examined categorical associations. McNemar’s test assessed dichotomous variable changes between groups. Student’s *t*-test compared parametric means, and paired t/Wilcoxon compared time periods. Mann-Whitney/Kruskal-Wallis compared non-parametric groups. Spearman’s correlation assessed quantitative variable associations. Linear regression predicted risk factors. Odds ratios (OR) were used for categorical outcomes. *P* ≤ 0.05 was counted significant.

## Results

### Descriptive analysis for different parameters before intervention among the studied patients

This study involved 75 children with a median age of 13 years (range, 6–18 years); 64% of them were male and 36% were female. The patients’ median disease duration was 12 years (range, 5–17.5 years). The first blood transfusion was administered at a median age of 6 months (range, 1.5–36 months), and the median frequency of blood transfusions was 20 (range, 12–24) per year. Regarding chelation therapy, it was initiated at a median age of 1 year (range, 0.5–5 years), with 96% of children demonstrating good compliance. Chelation therapy was administered in the form of deferasirox to all patients at a dose of 10–28 mg/kg/day, and deferoxamine was added to 28% of patients with severe iron overload at a dose of 40–50 mg/kg/day.

Of all the children, 8% presented with cardiac symptoms, including dyspnea, chest pain, dizziness, and syncope; 8% presented with thalassemic complications, including hepatitis C virus and diabetes mellitus; and 16% underwent splenectomy. The severity of thalassemia was classed as moderate in 88% of patients and severe in 12% of patients. With respect to the laboratory results, the median hemoglobin level was 9.1 (range, 8.0–10.30) g/dl. Serum ferritin level was < 2500 ng/ml in 52% of patients, between 2500 and 5000 ng/ml in 28% of patients and >5000 ng/ml in 20% of patients. As regards vitamin D levels, 18.7% of patients had insufficient vitamin D (20–29.9 ng/ml), while 81.3% had deficient levels (< 20 ng/ml).

### Association of vitamin D level with patients’ characteristics before intervention

Several key associations between vitamin D levels before intervention and demographic data, disease characteristics, laboratory measurements, and magnetic resonance imaging (MRI) findings are highlighted in Table 1.

**Table 1:** Association of vitamin D level with patients’ characteristics before intervention

Parameters		Vitamin D level				*p*-value
		Deficient (*n* = 61)		Insufficient (*n* = 14)		
		Mean ± SD	Range	Mean ± SD	Range	
*Demographic data and disease-related history*						
Age (years)		12.66 ± 3.79	7–18	9.79 ± 2.83	6–13	0.006*
Sex, *n* (%)	Male	34 (55.7%)		14 (100%)		0.002*
	Female	27 (44.3%)		0 (0%)		
*Disease characteristics*						
Disease duration (years)		11.46 ± 3.72	5–17.5	8.79 ± 2.85	5.5–12.5	0.016*
Age at onset of blood transfusion (months)		9.32 ± 7.45	1.5–36	10.5 ± 7.42	6–24	0.772
Frequency of blood transfusion (n/year)		19.38 ± 4.78	12–24	21.43 ± 3.08	18–24	0.138
Age at onset of chelation therapy (years)		1.53 ± 1.17	0.5–5	1.04 ± 0.57	0.5–2	0.121
Chelation type, *n* (%)	Deferasirox	40 (65.6%)		14 (100.0%)		0.008*
	Deferasirox + deferoxamine	21 (34.4%)		0 (0.0%)		
Cardiac symptoms, *n* (%)		6 (9.8%)		0 (0.0%)		0.586
Thalassemic complications, *n* (%)		6 (9.8%)		0 (0.0%)		0.586
Splenectomy, *n* (%)		12 (19.7%)		0 (0%)		0.020*
Thalassemic severity, *n* (%)	Moderate	52 (85.2%)		14 (100%)		0.195
	Severe	9 (14.8%)		0 (0%)		
*Laboratory measurements*						
Hemoglobin (g/dl)		9 ± 0.76	8–10.3	9.11 ± 0.4	8.2–9.5	0.393
WBCs (×10^3^/mm^3^)		10.69 ± 6.19	4.9–26.3	8.52 ± 2.93	4.53–11.72	0.663
Platelets (×10^3^/mm^3^)		362.8 ± 183.5	146–1014	295.6 ± 81.19	173–388	0.536
ALT (U/L)		52.46 ± 32.32	16–125	25.29 ± 6.39	15–30	< 0.001*
AST (U/L)		55.62 ± 26.53	22–115	30.0 ± 4.04	25–36	< 0.001*
Urea (mg/dl)		29.28 ± 8.10	15–47	33.71 ± 10.51	25–49	0.351
Creatinine (mg/dl)		0.62 ± 0.27	0.4–1.3	0.53 ± 0.2	0.3–0.9	0.225
Serum ferritin (ng/ml)		2894.3 ± 2173.5	200–6970	2472.3 ± 652.1	1500–3347	0.860
Ferritin, *n*(%)	< 2500	33 (54.1%)		6 (42.9%)		0.010*
	2500–5000	13 (21.3%)		8 (57.1%)		
	> 5000	15 (24.6%)		0 (0%)		
MRI	Heart (T2*) (ms)	29.79 ± 13.74	7.50–50.10	62.58 ± 25.22	34.94–81.0	0.011*
	Liver (LIC) (mg/g)	8.82 ± 5.37	1.22–16.40	5.58 ± 3.66	1.56–8.25	0.251

*SD* standard deviation, *WBCs* white blood cells, *ALT* alanine transaminase, *AST* aspartate transaminase, *MRI* magnetic resonance imaging, *LIC* liver iron concentration

^*^Significant when *p* value <0.05

Spearman correlation analysis between Vitamin D and baseline patients’ characteristics showed a strong negative correlations of vitamin D level with age (rs = −0.527, *p* < 0.001) and disease duration (rs = −0.453, *p* < 0.001) and substantial positive associations with onset of blood transfusion (rs = 0.26, *p* = 0.024) and onset of chelation therapy (rs = 0.284, *p *= 0.014).

### Association of vitamin D level with echocardiographic parameters before intervention

When comparing vitamin D deficient and insufficient groups regarding the parameters of the conventional echocardiography, only the deceleration time was considerably higher in the vitamin D deficient group than the insufficient group (*p* = 0.042). Regarding the STE parameters, the values of apical (AP) 4, 3, and 2 longitudinal (L) and the global longitudinal strains (GLS) were worse in the deficient than in the insufficient group (*p* = 0.034, 0.002, 0.027, and 0.005, respectively), as displayed in Table 2.

**Table 2:** Association of vitamin D level with echocardiographic parameters of left ventricle before intervention

Parameters	Vitamin D level				*p*-value
	Deficient (*n* = 61)		Insufficient (*n* = 14)		
	Mean ± SD.	Range	Mean ± SD.	Range	
*Conventional echocardiography*					
LVEDD (cm)	4.77 ± 0.56	3.39–5.60	4.53 ± 0.50	3.87–5.20	0.089
* Z* score	1.18 ± 0.97	−2.02–2.57	1.28 ± 0.56	0.43–2.0	0.703
Fractional shortening (%)	38.95 ± 3.23	32.80–45.20	41.24 ± 6.61	35.0–53.0	0.577
Ejection fraction (%)	69.05 ± 4.35	59.0–76.70	71.77 ± 6.28	66.0–83.0	0.698
E wave (c/s)	95.21 ± 11.97	76.60–121.0	100.4 ± 5.45	96.0–112.0	0.247
A wave (c/s)	60.41 ± 11.40	41.20–87.0	61.02 ± 5.97	52.90–71.90	0.698
E/A	1.61 ± 0.26	1.23–2.20	1.64 ± 0.10	1.54–1.80	0.230
Deceleration time (ms)	121.0 ± 15.33	98.0–144.0	110.1 ± 4.51	102.0–114.0	0.042*
SPAP (mmHg)	28.95 ± 6.81	20.0–45.0	28.50 ± 4.43	20.0–35.0	0.585
*Tissue Doppler imaging*					
S wave (c/s)	8.85 ± 1.72	6.27–12.40	8.69 ± 1.22	7.70–11.50	0.653
E wave (c/s)	13.07 ± 1.76	10.80–17.80	13.64 ± 1.98	12.30–18.50	0.340
A wave (c/s)	7.36 ± 1.29	4.70–9.40	7.21 ± 1.23	5.50–8.90	0.695
ICT (ms)	37.59 ± 6.93	26.0–56.0	36.64 ± 5.96	29.0–46.0	0.650
IRT (ms)	46.84 ± 8.50	29.0–61.0	45.07 ± 5.57	40.0–53.0	0.248
ET (ms)	275.5 ± 29.47	198.0–343.0	269.1 ± 20.76	243.0–296.0	0.440
MPI	0.31 ± 0.05	0.22–0.40	0.31 ± 0.04	0.27–0.37	0.495
E/E'	7.39 ± 1.30	5.26–9.46	7.44 ± 0.71	6.05–8.37	0.828
*Speckle tracking echocardiography*					
AP4 L Strain (%)	−21.99 ± 3.44	−27.50–−16.10	−24.31 ± 3.02	−28.20–−20.40	0.034*
AP3 L Strain (%)	−19.44 ± 3.81	−26.70–−12.40	−23.36 ± 3.94	−28.0–−16.70	0.002*
AP2 L Strain (%)	−20.0 ± 3.93	−26.70–−10.90	−22.07 ± 0.78	−22.90–−20.90	0.027*
GLS (%)	−20.40 ± 3.48	−26.40–−13.90	−23.22 ± 2.19	−26.20–−19.40	0.005*

*SD standard deviation, LVEDD* left ventricular end-diastolic diameter, *E wave* early passive filling of the left ventricle, *A wave* active filling of the atrial contraction, *E/A* E wave of conventional echocardiography/A wave of conventional echocardiography, *SPAP* systolic pulmonary artery pressure, *S wave* positive systolic wave represent myocardial contraction, *ICT* isovolumetric contraction time, *IRT* isovolumetric relaxation time, *ET* ejection time, *MPI* myocardial performance index, *E/E′* E wave of conventional echocardiography/E wave of tissue doppler, *AP2/3/4 L* apical two/three/four chamber view longitudinal strain, *GLS* global longitudinal strain

^*^Significant when *p* value < 0.05

On Spearman correlation analysis between vitamin D and conventional echocardiography, vitamin D level had an inverse relationship with left ventricular end-diastolic diameter (LVEDD) (rs = −0.403, *p* < 0.001) and deceleration time (rs = −0.338, *p* = 0.003). For the STE parameters, vitamin D level was positively correlated to the absolute value of GLS (rs = 0.438, *p* < 0.001).

### Comparison of vitamin D level and echocardiographic parameters before and after intervention

**Fig. 1:**
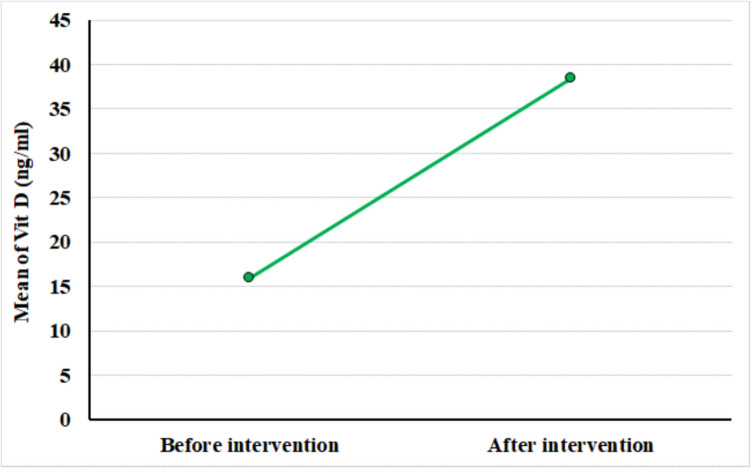
Line chart for vitamin D level before and after intervention

The mean Vitamin D level surged from 15.93 ± 3.61 (range, 9–22) ng/ml before intervention to 38.39 ± 3.95 (range, 31–45) ng/ml after intervention, showing a highly significant substantial improvement (*p* < 0.001) (Figure 1).

For the conventional and tissue Doppler echocardiography, the post-intervention data show significant improvement in multiple echocardiographic parameters. A noticeable improvement in STE parameters after vitamin D intervention is observed in Table 3.

**Table 3:** Comparison between left *ventricular* echocardiographic parameters before and after intervention

Parameters	Before intervention		After intervention		*p*-value
	Mean ± SD.	Range	Mean ± SD.	Range	
*Conventional echocardiography*					
LVEDD (cm)	4.80 ± 0.51	3.66–5.70	4.73 ± 0.56	3.39–5.60	<0.001*
* Z* score	1.20 ± 0.90	−2.02–2.57	1.03 ± 0.79	−1.25–2.43	<0.001*
Fractional shortening (%)	39.38 ± 4.11	32.80–53.0	40.66 ± 4.93	34.0–55.0	<0.001*
Ejection fraction (%)	69.56 ± 4.84	59.0–83.0	71.47 ± 5.43	60.0–85.0	<0.001*
E wave (c/s)	96.19 ± 11.21	76.60–121.0	100.4 ± 12.05	80.0–124.0	<0.001*
A wave (c/s)	60.53 ± 10.57	41.20–87.0	58.28 ± 10.72	40.50–86.0	<0.001*
E/A	1.62 ± 0.24	1.23–2.20	1.73 ± 0.27	1.40–2.40	<0.001*
Deceleration time (ms)	119.0 ± 14.58	98.0–144.0	111.9 ± 11.37	95.0–137.0	<0.001*
SPAP (mmHg)	28.87 ± 6.41	20.0–45.0	27.49 ± 5.18	20.0–40.0	<0.001*
*Tissue Doppler imaging*					
S wave (c/s)	8.82 ± 1.63	6.27–12.40	9.27 ± 1.56	6.60–12.50	<0.001*
E wave (c/s)	13.18 ± 1.80	10.80–18.50	14.37 ± 2.20	11.70–19.50	<0.001*
A wave (c/s)	7.33 ± 1.27	4.70–9.40	6.89 ± 1.14	4.70–9.30	0.001*
ICT (ms)	40.20 ± 6.73	26.0–53.0	37.41 ± 6.73	26.0–56.0	<0.001*
IRT (ms)	46.59 ± 6.88	34.0–60.0	46.51 ± 8.03	29.0–61.0	1.000
ET (ms)	272.4 ± 28.07	221.0–338.0	274.3 ± 28.05	198.0–343.0	0.431
MPI	0.32 ± 0.04	0.24–0.40	0.31 ± 0.05	0.22–0.40	0.001*
E/E'	7.40 ± 1.21	5.26–9.46	7.11 ± 1.35	4.90–9.70	<0.001*
*Speckle tracking echocardiography*					
AP4 L Strain (%)	−22.42 ± 3.47	−28.20–−16.1	−23.32 ± 3.36	−28.90–−16.5	<0.001*
AP3 L Strain (%)	−20.17 ± 4.11	−28.0–−12.40	−21.40 ± 3.75	−27.30–−14.0	<0.001*
AP2 L Strain (%)	−20.39 ± 3.64	−26.70–−10.9	−22.45 ± 4.14	−29.20–−12.2	<0.001*
GLS (%)	−20.92 ± 3.45	−26.40–−13.9	−22.45 ± 3.41	−27.20–−16.0	<0.001*

*SD standard deviation, LVEDD* left ventricular end-diastolic diameter, *E wave* early passive filling of the left ventricle, *A wave* active filling of the atrial contraction, *E/A* E wave of conventional echocardiography/A wave of conventional echocardiography, *SPAP* systolic pulmonary artery pressure, *S wave* positive systolic wave represent myocardial contraction, *ICT* isovolumetric contraction time, *IRT* isovolumetric relaxation time, *ET* ejection time, *MPI* myocardial performance index, *E/E′* E wave of conventional echocardiography/E wave of tissue doppler, *AP2/3/4 L* apical two/three/four chamber view longitudinal strain, *GLS* global longitudinal strain

^*^Significant when *p* value < 0.05

### Predictors of GLS impairment before intervention in β-TM children

The univariate linear regression analysis identified patient’s age, disease duration, and vitamin D deficiency as significant predictors of GLS impairment (*p* < 0.001, *p* < 0.001, and *p* = 0.005). However, in the multivariate analysis, the patient’s age remained the only strong predictor (*p* < 0.001), as shown in Table 4.

**Table 4:** Linear regression for prediction of GLS impairment before intervention

Parameter	Univariate		Multivariate	
	***β***	***p***	***β***	***p***
Deficient vitamin D	0.321	0.005*	0.122	0.159
Serum ferritin	0.138	0.238		
Age	0.703	< 0.001*	1.024	< 0.001*
Disease duration	0.635	< 0.001*	0.376	0.169
Severe thalassemic	−0.008	0.944		
Abnormal MRI (T2*)	0.207	0.273		
Abnormal MRI (LIC)	−0.086	0.651		

*β* standardized coefficients, *MRI* magnetic resonance imaging, *LIC* liver iron concentration

^*^Significant when *p* value < 0.05

## Discussion

Patients with β-TM are at risk of impaired cardiac function through many mechanisms: chronic anemia, which could lead to hyperdynamic circulation and elevated cardiac output [23]; iron overload, which leads to cardiac iron deposition and oxidative damage through the generation of reactive oxygen species, causing heart failure [24]**.** Moreover, deficits in specific nutrients, such as vitamin D, selenium, zinc, copper, and thiamine, could enhance cardiac impairment [25, 26]**.**

In this study, vitamin D levels were deficient in 81.3% of β-TM children. A recent systematic analysis of 12 studies found that the frequency of vitamin D deficiency varied from 24.8 to 80.6% [27]. Crucially, it has been proven that thalassemia patients exhibited lower vitamin D levels than matched healthy persons [28]**.** The association of vitamin D levels with several patients’ characteristics was assessed in the current work. There was a strong negative correlation between vitamin D levels and age and disease duration, proposing that the adverse effects of low vitamin D concentration increase with age and prolonged disease exposure.

Patients with high serum ferritin, lower heart T2* on MRI, on double chelation, with elevated liver enzymes, and those who had splenectomy were in the deficient group; these findings could refer to the association between vitamin D levels and disease severity and could suggest a potential link between vitamin D deficiency and myocardial iron deposition. It has been demonstrated that vitamin D deficiency increases cardiac iron uptake through promoting transmembrane calcium transport via left ventricular-dependent calcium channel (LVDCC), resulting in cardiomyopathy [29, 30]. In a prior study, myocardial iron overload was significantly higher with vitamin D insufficiency (eight folds) and deficiency (twenty folds) [8]**.**

In the current work, STE revealed significant differences in LV longitudinal strains and GLS between vitamin D-deficient and insufficient groups, with correlations suggesting that vitamin D levels inversely affect LVEDD and positively influence GLS, impacting systolic and diastolic functions. The findings of this investigation align with those of earlier research, as a positive correlation between vitamin D levels and LV function was previously reported [19, 26], and vitamin D concentrations were found to be related to both LV diastolic functions and LV ejection fraction [25, 31].

In our study, noticeable improvements in STE parameters were recorded after vitamin D supplementation. STE changes were all statistically significant with *p* < 0.001, indicating substantial enhancements in myocardial function. These improvements indicate better myocardial performance following intervention. The results highlight the potential cardioprotective effects of maintaining adequate vitamin D levels. Comparable findings were reported previously, as prior to vitamin D treatment; STE revealed a much lower absolute value of the LV GLS. Following vitamin D replenishment, parameters substantially increased indicating that left ventricular systolic and diastolic functions increased significantly (*p* < 0.05) [31].

Finally, when univariate linear regression was applied to find the important predictors of GLS impairment, vitamin D deficiency showed a positive connection, suggesting that lower vitamin D levels were linked with reduced GLS; another significant predictor was age, indicating that a lower GLS is linked to older age. In the multivariate analysis, age continued to be a powerful predictor.

This is consistent with another study that used multivariate regression analysis to find that vitamin D levels were an independent predictor of cardiac T2* values. This implies that assessments of vitamin D levels ought to be employed as a risk assessment tool for myocardial iron overload in clinical settings. They set a vitamin D cut-off value of 17.3 ng/dL to assess if patients were at higher risk for myocardial iron overload. They recommended that vitamin D therapy should be initiated or increased in these patients to avoid future cardiac iron buildup. They emphasized that while age, length of illness, and low vitamin D levels are important factors, age may be the most important predictor of GLS when other factors are considered [8].

This is the first study, as far as we know, to employ STE in Egypt to assess how vitamin D replacement affects LV in β-TM children. However, we were incapable of comparing vitamin D-deficient patients with those who had normal vitamin D levels, as we did not find any; this is one of the limitations of our study. We did not use heart T2* magnetic resonance imaging to determine cardiac iron overload before and after the supplement to estimate the effect of vitamin D supplementation on cardiac iron overload, due to its cost and limited availability. Our study did not assess how other nutritional deficits affected cardiac function. In fact, many evaluations of vitamin D levels and LV GLS throughout time may support our conception.

## Conclusion

Vitamin D deficiency is common in β-TM children and has been found to be correlated with impairment of both systolic and diastolic cardiac function as examined by STE. Additionally, patients with β-TM can benefit from adequate vitamin D substitution to improve their cardiac functions. Therefore, we recommend that serum vitamin D concentration of these patients should be regularly monitored and corrected in order to prevent consequences from vitamin D deficiency.

## Data Availability

ALL data is provided within the manuscript

## Abbreviations

25-OH vitamin D: 25-hydroxyvitamin D
AP: Apical
CLIA: Chemiluminescent immunoassay
ELISA: Enzyme-linked immunosorbent assay
GLS: Global longitudinal strain
L: Longitudinal
LV: Left ventricular
LVDCC: Left ventricular-dependent calcium channel
LVEDD: Left ventricular end-diastolic diameter
MRI: Magnetic resonance imaging
OR: Odds ratios
PTH: Parathyroid hormone
SD: Standard deviation
SPSS: Statistical Package for the Social Sciences
STE: Speckle tracking echocardiography
